# Relationship of immunonutritional factor with changes in liver volume after portal vein embolization^☆^

**DOI:** 10.1016/j.sopen.2022.05.012

**Published:** 2022-05-28

**Authors:** Atsushi Nanashima, Yukinori Tanoue, Koichi Yano, Masahide Hiyoshi, Naoya Imamura, Takeomi Hamada, Kengo Kai, Eiji Kitamura, Yasuto Suzuki, Kousei Tahira, Fumiya Kawano, Takeshi Nagayasu

**Affiliations:** a Division of Hepato-Biliary-Pancreas Surgery, Department of Surgery, University of Miyazaki Faculty of Medicine, University of Miyazaki Hospital, Kihara Kiyotake, Miyazaki 889-1692, Japan; b Division of Surgical Oncology, Nagasaki University School of Biomedical Sciences, 1-7-1 Sakamoto, Nagasaki 852-8501, Japan

## Abstract

**Background:**

To identify predictors of changes in hepatic volumes after portal vein embolization, we examined the relationship with preoperative nutritional and immunological parameters.

**Patients and Methods:**

Ninety-three patients who underwent portal vein embolization were included. The control group comprised 13 patients who underwent right hepatectomy without portal vein embolization. Computed tomographic volumetric parameter was measured for changes in embolized and nonembolized liver. Correlation with various candidates of immunonutritional parameters was examined.

**Results:**

Difference in increased liver ratio was 9.1%. C-reactive protein levels significantly increased after portal vein embolization (P < .01), whereas albumin and total cholesterol levels significantly decreased, respectively (P < .01). The C-reactive protein/albumin ratio, prognostic nutritional index, Controlling Nutritional Status score, and modified Glasgow Prognostic Score were significantly different, respectively (P < .01). Prothrombin activity and total cholesterol level significantly correlated with the increased change in nonembolized liver (P < .05). The C-reactive protein and C-reactive protein/albumin ratio after portal vein embolization negatively correlated with hypertrophic ratio (P < .05). By comparing posthepatectomy outcomes between 64 patients undergoing portal vein embolization and 13 who did not, the prevalence of severe complications and mortality in the portal vein embolization group was not different from that in the non–portal vein embolization group. Liver activity at 15 minutes > 0.92 and increased liver volume ≥ 10% tended to correlate with lower prevalence of severe complications. Only increased intraoperative blood loss ≥ 1,500 mL was significantly associated with morbidity and mortality (P < .05).

**Conclusion:**

Contrary to our hypothesis, immunonutritional parameters, except C-reactive protein and C-reactive protein/albumin ratio, did not reflect hypertrophy after portal vein embolization. Although it is difficult to predict the hypertrophic degree, the strategy of scheduled hepatectomy should be switched in case of impaired inflammatory status after portal vein embolization.

## INTRODUCTION

The operative morbidity or mortality of patients who underwent major hepatectomy has significantly decreased recently with adequate preoperative assessment and strategy of hepatic function, precise estimation of liver volume, and improvements in perioperative managements [1,2]. However, the risk of posthepatectomy risks in patients with coexisting impaired nutritional or immunological functions still remains [3,4]. In patients undergoing major hepatectomy or more extensive liver resection of more than 60% of the entire liver parenchyma, preoperative portal vein embolization (PVE), which allows a smaller hepatic resection and induces hypertrophy of the remnant liver, has become a useful and safe standard option. Other embolization procedures such as associating liver partition and PVE for staged hepatectomy have been attempted as well [[5], [6], [7]]. PVE can extend the operative indications and improve posthepatectomy prognosis [5,6,8]. The clinical significance of PVE has been well recognized, and it is often incorporated into the preoperative preparation for major hepatectomy. However, in previous studies including our reports, preoperative predictive parameters for hepatic volume hypertrophy in the nonembolized liver have not been fully elucidated [[8], [9], [10]].

Preoperative nutritional, immunological, or their combined parameters might be associated with posthepatectomy complications and patient prognosis [1,2,[11], [12], [13], [14]]. These patient factors were also related to organ regeneration or wound healing [15,16]. Therefore, better regeneration of nonembolized liver after PVE and hepatectomy may prevent posthepatectomy-related complications such as hepatic failure. The relationship between nutritional or immunological parameters and increased nonembolized liver volume after PVE has not yet been clarified, and correlation between immunonutritional factors and hypertrophy of the nonembolized lobe has not been fully elucidated by our previous study yet [17]. Thus, we hypothesized in the present study that the conceivable parameters or score using nutritional and immunological parameters might closely reflect a correlation with morphological hepatic volume, which can be predictive of the PVE effect.

In the present study, we examined the patient clinical or liver functional parameters, the reported immunonutritional parameters, and changes in hepatic volume of the nonembolized liver after PVE for major hepatectomy in patients with various malignancies at 2 academic institutes to improve the clinical strategy before major hepatectomy.

## PATIENTS AND METHODS

### Patients

This retrospective study collected data of 106 consecutive patients with various liver malignancies at the Division of Surgical Oncology, Department of Translational Medical Sciences, Nagasaki University Graduate School of Biomedical Sciences (NUGSBS), between April 2006 and March 2015 (*n* = 30) and the Division of Hepatobiliary Pancreatic Surgery, Department of Surgery, University of Miyazaki Faculty of Medicine (UoM), between January 2008 and September 2021 (*n* = 76), which are managed by the first author at present. The data of 13 of 106 patients served as control data, and these were compared with those of the remaining 93 patients who underwent PVE. All patients' in-hospital data were retrospectively and consecutively collected from patient charts at the 2 institutions. The study design was approved by the Ethics Review Board of NUGSBS and UoM (assigned as #21122008, December 27, 2021, and #O-1034, October 25, 2021, respectively). Informed consent was obtained via an opt-out procedure at an outpatient clinic and on our website information for a month. No financial support was received for this study, and the authors declare no conflicts of interest. Data were retrieved from both anesthetic and patient electric charts plus the NUGSBS and UoM database for the duration of the initial hospitalization following the hepatectomy.

In our hospital, the volume of the liver to be resected is determined preoperatively based on the indocyanine green retention rate at 15 minutes (ICGR15) using Takasaki's formula [18]. The estimated resected liver volume, excluding tumor volume (cm^3^), is measured using computed tomography (CT) volumetry [19]. In cases where the permitted resected volume of the liver is greater than the estimated resected volume of the liver, the planned hepatectomy is performed. In cases where the permitted resected volume is less than the estimated volume or the estimated volume is more than 65% of the normal liver and more than 50% of a cirrhotic liver, preoperative PVE is selected [20]. Liver activity at 15 minutes (LHL15) on ^99m^Tc-galactosyl serum albumin (GSA) scintigraphy was performed preoperatively with ICGR15 [20].

### Portal Vein Embolization and Evaluation

The 2 approaches to the right portal vein were direct catheterization of the ileocolic vein and percutaneous transhepatic puncture [5,6,8,9,20]. From 1999 to 2007, the substances used for embolization at NUGSBS included 1 g of absorbable gelatin sponge powder (Gelfoam; Upjohn, Kalamazoo, MI) and 5,000 U (5 mL) of liquid thrombin (Sankyou Co, Tokyo, Japan) mixed in the contrast media, or iodized oil (Lipiodol; Savage Lab, Melville, NY) mixed with gelatin (Sponzel; Astellas Pharma, Inc, Tokyo, Japan). Since 2008, a liquid embolization material and 5% of ethanolamine oleate iopamiodole (EOI; Oldamin; Takeda Pharma, Osaka, Japan) have been mainly used for embolization. Permanent embolization materials or coils are not used. In case of transiliac vein portal embolization (*n* = 4), the laparoscopic staging examination was simultaneously performed. At UoM, the mixed substances used for embolization included 1 g of absorbable gelatin sponge powder (Gelfoam; Upjohn, Kalamazoo, MI) and iodized oil (Lipiodol; Savage Lab., Melville, NY), and subsequent intravascular coil embolization was added since 2008 [21]. Transiliac vein portal embolization with staging laparoscopy was also performed in 2 cases since 2015. Embolization was completed when the entire right portal vein was occluded. Fourteen or 21 days after PVE, the hepatic volumes of the nonembolized lobe and embolized lobe (lobe to be resected) were reassessed using CT volumetry (CT vol) and ^99m^Tc-GSA scintigraphy [20]. Scheduled hepatectomy was usually performed 21–28 days after PVE.

### Compared Parameters

The conventional clinicopathological parameters analyzed were patient demographics (age, sex, body mass index per height and body weight, background liver disease), liver functional data (serum levels of hyaluronic acid [HA], bilirubin, alanine transaminase, prothrombin activity, platelet count, ICGR15, LHL15 on ^99m^Tc-GSA scintigraphy, substance used in embolization), and surgical records (curability, type of hepatectomy, intraoperative blood loss, posthepatectomy complication and its International Study Group [ISGPS] grade, 90-day hospital mortality). The serum HA level was measured using the sandwich binding protein assay by SRL, Inc (Tokyo, Japan), and the manufacturer's normal value of less than 50 ng/mL was used [21]. A dose of 0.5 mg ICG/kg body weight was injected intravenously, and the 15-minute retention rate was measure with a photopiece applied to the fingertip (Sumitomo Electric, Tokyo, Japan) at NUGSBS. The blood sampling method 5, 10, and 15 minutes after injection was used at UoM [22].

The main subjects of nutritional and immunological data were white blood cell count, lymphocyte count, red blood cell distribution width (RDW) [13], C-reactive protein (CRP), serum albumin, and total cholesterol levels and their combined immunonutritional scores (neutrophil–lymphocyte ratio [23], platelet–lymphocyte ratio [24], CRP–albumin ratio [25], Onodera's prognostic nutritional index [PNI] [26], Controlling Nutritional Status [CONUT] score [12], and the modified Glasgow Prognostic Score [mGPS] [27]), which were previously reported as significant predictive scores for digestive surgeries. These parameters were compared in relation to changes in CT hepatic volumes following PVE described below and the surgical record of patients who could proceed with the scheduled hepatectomy. The above data were analyzed before and 2 weeks after PVE. To assess posthepatectomy outcomes, the Clavien–Dindo classification grade and in-hospital mortality were determined [28].

### Volumetric Measurement Using CT

The morphological volume was measured using the contrast CT volume parameter [5,6,8,9,17]. Serial axial scans were taken at 3-mm intervals using a 16-row multidetector CT scanner, and the hepatic volume of areas without tumors and large vessels in each liver region was measured using Workstation software (Ziostation version 1.1, Ziosoft Inc, Tokyo, Japan, at NUSGBS and Synapse Vincent version 5.2, Fujifilm Co, Tokyo, Japan, at UoM). Regarding ^99m^Tc-GSA liver scintigraphy, all patients received 3 mg (185 MBq) of ^99m^Tc-GSA (Nihon Medi-Physics, Nishinomiya, Japan) as a bolus dose into the antecubital vein. Images were obtained with a large field-of-view gamma camera (Picker Prism-2000, Picker Prism International, Cleveland, OH) equipped with a high-resolution, parallel-hole collimator centered on the liver and the precordium. Sequential abdominal digital images (128 × 128 matrixes) were acquired with an online nuclear data processor (Odyssey Series, Picker Prism International) at 30 s/frame for the first 16 minutes after the injection.

Changes in hepatic volume were compared between pre- and post-PVE (2 weeks after PVE), and the changed rate and rate of actual volume were calculated.

### Statistical Analysis

Continuous data are expressed as mean ± SD. Data for different groups were compared using 1-way analysis of variance. The *χ*^2^ test was used for comparison of categorical variables. Differences between groups were analyzed using Fisher exact test or Scheffe multiple comparison test. Correlations between 2 parameters were examined by calculating Pearson correlation coefficient. The 95% confidence intervals for each correlation were calculated. The Statistical Package for the Social Sciences version 22.0 software (SPSS, Chicago, IL) was used for all statistical analyses.

## RESULTS

### Patient Demographics

The 93 participants who underwent PVE comprised 67 men and 26 women with a mean age (± SD) of 68.1 ± 9.3 years (range, 31–84 years). The liver diseases present were hepatocellular carcinoma (*n* = 15), intrahepatic cholangiocarcinoma (*n* = 9), metastatic liver carcinoma (*n* = 10), gallbladder carcinoma (*n* = 16), bile duct carcinoma (*n* = 41), leiomyosarcoma of vena cava (*n* = 1), and direct invasion of renal cell carcinoma (*n* = 1). The background hepatic condition was normal liver function (*n* = 27), chronic viral liver disease (*n* = 21 including cirrhosis in 7), obstructive jaundice (*n* = 42), chemotherapy associated liver injury (*n* = 2), and nonalcoholic steatohepatitis (*n* = 1). The extent of embolized liver was right hemiliver in 83 patients (89%), right trisections in 3, left hemiliver in 2, and left trisections in 5. The procedures of PVE were percutaneous transhepatic approach in 88 (94%), transiliac vein approach in 4, and percutaneous transhepatic portal vein embolization with subsequent transarterial chemoembolization in 1. No serious morbidity or mortality was recorded after PVE. Scheduled hepatectomy was successful in 64 patients (68%) and failed in 29 including 12 with preoperatively unresectable liver (abandoned owing to low estimated volume of the remnant liver preoperatively) and 17 with open laparotomy (abandoned owing to distant metastasis in 16 and pancreaticoduodenectomy alone in 1). The extent of hepatectomy in 64 patients who underwent hepatectomy was hemihepatectomy in 61 patients (95%) and trisectionectomy in 3. Additional operative procedures were extrahepatic bile duct resection in 22, pancreaticoduodenectomy in 5, the combined contralateral portal vein resection with anastomosis in 4, splenectomy in 2, and adrenectomy in 1. Intraoperative blood loss in hepatectomy was 1,852 ± 1,618 mL (range, 270–6,700 mL). Postoperative hepatectomy-related complications of ISGPS grade > IIIa occurred in 24 patients (26%) and in-hospital mortality in 6 patients (7%). The preoperative modified GPS was 0 for 52 (56%), 1 for 27 (29%), and 2 for 14 (15%) patients, respectively.

The control group (*n* = 13) comprised 9 men and 4 women with a mean age (± SD) of 62.2 ± 14.6 years (range, 39–86 years). The liver diseases present were hepatocellular carcinoma (*n* = 6), intrahepatic cholangiocarcinoma (*n* = 1), metastatic liver carcinoma (*n* = 3), gallbladder carcinoma (*n* = 1), and bile duct carcinoma (*n* = 2). The background hepatic condition was normal liver function (*n* = 4), chronic viral liver disease (*n* = 3 including compensatory cirrhosis in 2), obstructive jaundice (*n* = 3), chemotherapy associated liver injury (*n* = 2), and nonalcoholic steatohepatitis (*n* = 1). Scheduled hepatectomy was successful in all 13 patients, and the extent of hepatectomy was hemihepatectomy in 12 patients (93%) and trisectionectomy in 1. Additional operative procedures were extrahepatic bile duct resection in 1 and portal vein tumor thrombectomy in 1. Intraoperative blood loss in hepatectomy was 1,640 ± 994 mL (range, 300–3,600 mL). Postoperative hepatectomy-related complications of ISGPS grade > IIIa occurred in 2 patients (15%), but no mortality occurred. The preoperative modified GPS was 0 for 6 (46%), 1 for 5 (39%), and 2 for 2 (15%) patients. There were no significant differences in each parameter between groups.

### CT Volume Parameters and Liver Function

In 90 participants who underwent PVE of the right liver, the CT volume parameters estimated before and after PVE were 1,372 ± 340 and 1,354 ± 315 cm^3^, respectively, which were not significantly different (*P* = .63). The right hemiliver volume before and after PVE was 865 ± 250 (62.4% ± 9.5%) and 713 ± 230 (52.9% ± 10.2%) cm^3^, respectively. The embolized liver volume was significantly lower than that before (*P* < .0001). The differences in decreased liver volume and percentage were 85 ± 218 cm^3^ and 9.1% ± 8.8%, respectively. The left hemiliver volumes before and after PVE were 505 ± 186 and 643 ± 207 cm^3^, respectively. The embolized liver volume was significantly lower than that before (*P* < .0001). The differences in increased liver volume and percentage were 143 ± 136 cm^3^ and 9.1% ± 8.8%, respectively. In the 3 patients who underwent PVE of the trisegment, the CT volume parameters estimated before and after PVE were 1,299 ± 348 and 1,282 ± 420 cm^3^, respectively, which were not significantly different (*P* = .75). The embolized trisegment-liver volume before and after PVE was 988 ± 394 (74.3% ± 10.6%) and 721 ± 194 (57.39 ± 6.1%) cm^3^, respectively, which were not significantly different (*P* = .25). The differences in decreased liver volume and changed percentage were 267 ± 200 cm^3^ and 17% ± 14.7%, respectively. The nonembolized liver volumes before and after PVE were 311 ± 79 cm^3^ and 561 ± 233 cm^3^, respectively, which were not significantly different (*P* = .5). The difference in increased liver volume was 247 ± 262 cm^3^.

In all 93 patients who underwent PVE, changes in serum total bilirubin level before and after PVE were 2.1 ± 3.1 and 1.2 ± 1.7 mg/dL (*P* = .0002), changes in alanine transferase (ALT) level before and after PVE were 63.4 ± 69.1 and 53.5 ± 50.2 U/L (*P* = .32), changes in prothrombin activity before and after PVE were 88.8% ± 18.4% and 85.3% ± 13.8% (*P* = .0014), and changes in platelet count before and after PVE were 22.5 ± 7.6 × 10^4^ and 23.4 ± 8.8 × 10^4^/mm^3^ (*P* = .65), respectively. After PVE, the total bilirubin level significantly decreased, and prothrombin activity significantly but slightly decreased. Changes in serum HA level before and after PVE were 125 ± 187 and 136 ± 184 ng/mL (*P* = .08), changes in ICGR15 before and after PVE were 11.2% ± 6.5% and 12.2% ± 7.2% (*P* = .072), and changes in liver uptake ratio (LHL15) on ^99m^Tc-GSA scintigraphy 3–15 minutes before and after PVE were 0.914 ± 0.041 and 0.910 ± 0.039 (*P* = .06), respectively. There were no significant differences in liver functional reserve parameters after PVE. In the surgical records, the blood loss in 64 patients who underwent scheduled hepatectomy was 1,851 ± 1,617 mL. Postoperative complications of ISGPS grade > IIIa occurred in 22 patients (35%), and 4 mortalities (6.3%) were recorded. Postoperative liver failure was observed in 8 patients.

In the 13 patients who underwent right hepatectomy without PVE, the total liver volume was 1,145 ± 204 cm^3^; the right liver volume was 624 ± 152 cm^3^ (54.7% ± 8.4%); the left liver volume was 511 ± 121 cm^3^; the total bilirubin level was 0.63 ± 0.25 mg/dL; the alanine transaminase level was 46.6 ± 21.5 U/L; the prothrombin activity was 77.8% ± 36.7%; the platelet count was 26.9 ± 10.3 × 10^4^/mm^3^; the serum hyaluronic level was 73 ± 47 ng/mL; the ICGR15 was 9.3% ± 4.9%; and the LHL15 was 0.930 ± 0.028. The total liver volume (− 211 cm^3^) and the right liver volume (− 227 cm^3^) or ratio (− 7.9%) were significantly lower in the non-PVE group (*P* < .05). However, the left liver volume was not significantly different between the groups (*P* = .39). Concerning the liver function parameters, the pre-PVE total bilirubin in the non-PVE group was significantly lower than that in the PVE group (*P* = .010).

In the surgical records, the blood loss in 13 patients was 1,640 ± 994 mL, which was not significantly different from that in patients who underwent PVE (*P* = .89). Postoperative complications of ISGPS grade > IIIa occurred in 2 patients (15%), and there were no mortalities. These were not significantly different when compared with findings of patients who underwent hepatectomy after PVE. Postoperative liver failure was not observed.

By comparing patient outcomes between the 64 patients who underwent preoperative PVE and those who did not, the prevalence of postoperative complications of Clavien–Dindo > IIIa and in-hospital mortality in the PVE group (35% and 6%) was not significantly different when compared with that in the non-PVE group (15% and 0%) (*P* = .292 and .999, respectively). In all the 77 patients, Clavien–Dindo grade IIIa was observed in 1 patient who underwent right trisectionectomy, 21 who underwent right hepatectomy, and 1 who underwent left trisectionectomy.

### Preoperative Immunonutritional Parameters and Scores

In the 93 patients who underwent PVE, changes in white cell count before and after PVE were 6,082 ± 2,069 and 6,362 ± 2,940/mm^3^ (*P* = .58); changes in the lymphocyte ratio before and after PVE were 27% ± 11% and 25% ± 10% (*P* = .19); changes in the neutrophil–lymphocyte ratio (neutrophil count/lymphocyte count) before and after PVE were 3.65 ± 2.30 and 4.09 ± 3.66 (*P* = .42); changes in the platelet–lymphocyte ratio (platelet count/lymphocyte count) before and after PVE were 0.017 ± 0.001 and 0.018 ± 0.011 (*P* = .84); changes in RDW before and after PVE were 14.8 ± 2.1 and 14.7 ± 1.7 (*P* = .16); and changes in CRP before and after PVE were 0.95 ± 1.66 and 1.61 ± 2.70 mg/dL (*P* = .003), respectively. CRP significantly increased after PVE. With respect to nutritional parameters, changes in albumin before and after PVE were 3.61 ± 0.55 and 3.29 ± 0.52 mg/dL (*P* < .001); changes in total cholesterol before and after PVE were 202 ± 76 and 168 ± 53 mg/dL (*P* < .001); changes in the CRP–albumin ratio (CRP/ albumin) before and after PVE were 0.31 ± 0.58 and 0.53 ± 0.93 (*P* = .0011); changes in PNI before and after PVE were 43.7 ± 7.4 and 40.3 ± 5.9 (*P* < .001); changes in CONUT scores before and after PVE were 2.78 ± 2.19 and 3.88 ± 2.49 (*P* < .001); and changes in modified GPS before and after PVE were 0.59 ± 0.74 and 0.97 ± 0.79 (*P* < .001). After PVE, the serum albumin, total bilirubin level, and PNI significantly decreased, and the CRP–albumin ratio, CONUT score, and modified GPS significantly increased.

### Correlation Between Changes in Liver Volume and Each Parameter

Table 1 shows the relationship between hepatic volume and various liver function and immunonutritional parameters. Sex, background liver disease, and embolization procedures were not significantly related to changes in hepatic volumes or the hypertrophic ratio of nonembolized liver after PVE. Table 2 shows the correlation between the continuous parameters and changes in liver volumetric parameters after PVE. The LHL15, height, and total bilirubin level before PVE significantly correlated with the decrease in embolized liver volumes (*P* < .05). The preoperative prothrombin activity and total cholesterol level significantly correlated with increased change in nonembolized liver and its increased percentage (*P* < .05). The other immunonutritional parameters were not significantly correlated with any change in liver volumes.

**Table 1:** Relationship between changes in hepatic volume after portal vein embolization and clinicopathological parameters

	*Liver volume*			*Hypertrophic ratio*
	*Whole liver (cm*^*3*^*)*	*Embolized liver (cm*^*3*^*)*	*Nonembolized liver (cm*^*3*^*)*	*Nonembolized liver (%)*
Sex				
Male (*n* = 67)	− 4.9 ± 217	− 94 ± 202	146 ± 140	8.4 ± 9.4
Female (*n* = 26)	− 52 ± 236	− 83 ± 263	145 ± 145	11.9 ± 7.8
Background liver				
Normal (*n* = 27)	44 ± 304	− 53 ± 258	130 ± 167	6.8 ± 11.5
Fatty (*n* = 1)	548	− 303	79	9.1
Chemotherapy associated injury (*n* = 2)	− 87 ± 87	14.7 ± 94	35 ± 44	2.0 ± 2.8
Chronic viral hepatitis (*n* = 14)	− 40 ± 96	− 63 ± 132	143 ± 120	10.6 ± 7.6
Cirrhosis (*n* = 7)	− 38 ± 171	− 58 ± 235	118 ± 128	7.2 ± 10.0
Obstructive jaundice (*n* = 42)	23 ± 189	130 ± 218	168 ± 136	11.3 ± 11.5
Embolized area				
Right liver (*n* = 83)	− 4.8 ± 221	− 87 ± 211	150 ± 137	9.4 ± 9.1
Left liver (*n* = 2)	− 253 ± 235	− 150 ± 465	45 ± 22	6.5 ± 0.7
Right trisegment (*n* = 3)	− 31 ± 265	112 ± 134	85 ± 126	3.8 ± 4.9
Left trisegment (*n* = 5)	− 140 ± 248	− 262 ± 246	159 ± 232	13.4 ± 11.9
Route of embolization				
PTPE (*n* = 88)	− 4.7 ± 213	− 100 ± 210	149 ± 144	9.4 ± 9.3
TIPE (*n* = 4)	− 327 ± 262	74 ± 395	90 ± 46	10.5 ± 2.6
TAE followed by PVE (*n* = 1)	28	16	44	3
Embolization substance				
Gelatin fragment† + coil (*n* = 59)	− 16 ± 229	− 16 ± 229	170 ± 154	10.7 ± 9.7
Iodized oil‡ + Sponzel fragment (*n* = 9)	35 ± 286	− 130 ± 139	112 ± 159	3.8 ± 11.8
EOI (*n* = 25)	− 51 ± 182	108 ± 165⁎	97 ± 76	8.6 ± 4.7
Resectability				
Scheduled hepatectomy (*n* = 65)	− 47 ± 212	− 101 ± 235	141 ± 128	10.0 ± 8.5
Unresectable (*n* = 28)	45 ± 235	− 68 ± 183	156 ± 167	7.9 ± 10.2
Modified GPS				
0 (*n* = 52)	− 17 ± 209	− 101 ± 212	140 ± 126	10.3 ± 7.6
1 (*n* = 27)	− 8 ± 214	− 47 ± 225	149 ± 124	8.2 ± 10.3
2 (*n* = 14)	− 42 ± 294	− 137 ± 239	159 ± 218	8.2 ± 11.8

Data are presented as mean ± SD. *PTPE*, percutaneous transhepatic portal vein embolization; *TIPE*, transiliac vein portal embolization; *TAE*, transarterial chemoembolization.

⁎ *P* < .05 vs. others

† Sponzel (Astellas Pharma, Inc, Tokyo, Japan).

‡ Lipiodol (Savage Lab, Melville, NY).

**Table 2:** Correlation between the continuous parameters and changes in the CT volumetric parameter after PVE

	*Whole liver volume (cm*^*3*^*)*	*Decrease in embolized liver volume (cm*^*3*^*)*	*Increase in nonembolized liver volume (cm*^*3*^*)*	*Changes in hypertrophic ratio (%)*
	γ	Γ	γ	Γ
Age	0.153	− 0.105	0.018	− 0.017
Hyaluronate (ng/mL)	0.009	0.069	0.200	− 0.185
ICGR15 (%)	0.012	− 0.009	− 0.006	− 0.016
LHL15	0.004	− 0.237⁎	− 0.047	0.002
Height (cm)	− 0.010	0.567⁎⁎	0.187	0.186
Weight (kg)	− 0.110	0.195	− 0.056	− 0.012
Total bilirubin (mg/dL)	− 0.182	0.321⁎⁎	0.090	− 0.166
Alanine aminotransferase (IU/L)	0.053	0.065	− 0.020	− 0.051
Prothrombin activity (%)	0.066	0.117	0.319⁎⁎	− 0.217⁎
Lymphocyte count (/mm^3^)	0.091	0.015	0.016	− 0.054
Lymphocyte/white blood cell (%)	− 0.150	0.080	− 0007	− 0.102
Neutrophil/lymphocyte ratio	− 0.134	− 0.197	− 0.125	0.160
Platelet count (/mm^3^)	− 0.060	0.089	0.040	0.142
Platelet/lymphocyte ratio	− 0.071	− 0.027	0.005	0.045
RDW	0.077	0.135	0.171	− 0.049
Albumin (g/dL)	0.019	− 0.188	− 0.099	0.038
Total cholesterol (mg/dL)	− 0.077	0.196	0.238⁎	0.266⁎
CRP (mg/dL)	− 0.065	0.040	− 0.065	0.189
CRP/albumin ratio	− 0.095	0.082	− 0.071	0.157
Onodera's PNI	0.063	− 0.157	− 0.082	0.026
CONUT score	− 0.110	0.150	0.029	− 0.004
modified GPS	− 0.025	0.006	0.048	0.103

⁎ *P* < .05.

⁎⁎ *P* < .01.

Table 3 shows the differences of each parameter before and after PVE. The total bilirubin level significantly decreased (improved) 2 weeks after PVE (*P* < .01). The prothrombin activity, serum albumin level, total cholesterol level, and Onodera's PNI significantly decreased 2 weeks after PVE (*P* < .01), whereas the CRP level, CRP/albumin ratio, and CONUT classification score significantly increased 2 weeks after PVE (*P* < .01). The modified GPS score 1 or 2 weeks after PVE was significantly higher than that before PVE (*P* < .01). The correlation with each volumetric changes after PVE is indicated in Table 4. The CRP and CRP/albumin ratio 2 weeks after PVE negatively correlated with the hypertrophic ratio of nonembolized liver (*P* < .05). The other nutritional parameters did not show any correlation.

**Table 3:** Differences between the parameters before and 2 weeks after PVE

	*Before PVE*	*2 wk after PVE*	P *value*
Hyaluronate (ng/mL)	125 ± 187	136 ± 184	.080
ICGR15 (%)	11.2 ± 6.5	12.2 ± 7.2	.072
LHL15	0.913 ± 0.042	0.910 ± 0.039	.056
Total bilirubin (mg/dL)	2.1 ± 3.1	1.2 ± 1.7	<.01
Alanine aminotransferase (ALT) (IU/L)	63 ± 69	61 ± 84	.429
Prothrombin activity (%)	88 ± 18	85 ± 14	<.01
Platelet count (10^4^/mm^3^)	22.4 ± 7.6	23.3 ± 8.8	.645
Lymphocyte ratio (%)	26.9 ± 11.2	25.3 ± 9.5	.150
Lymphocyte count (/mm^3^)	1562 ± 775	1470 ± 580	.149
Neutrophil/lymphocyte ratio	3.7 ± 2.3	4.2 ± 3.5	.336
Platelet/lymphocyte ratio	0.017 ± 0.010	0.018 ± 0.011	.780
RDW	14.7 ± 2.1	14.7 ± 1.7	.162
Albumin (g/dL)	3.61 ± 0.54	3.29 ± 0.51	<.01
Total cholesterol (mg/dL)	202 ± 76	168 ± 53	<.01
CRP (mg/dL)	0.95 ± 1.65	1.65 ± 2.72	<.01
CRP/albumin ratio	0.31 ± 0.57	0.54 ± 0.93	<.01
Onodera's PNI	43.7 ± 7.3	40.2 ± 5.9	<.01
CONUT	2.80 ± 2.20	3.95 ± 2.54	<.01
Modified GPS (0/1/2)	52/28/13	30/36/27	<.01

**Table 4:** Correlation between parameters at 2 weeks and changes in the CT volume parameter after PVE

	*Changes of whole liver volume (cm*^*3*^*)*	*Decrease in embolized liver volume (cm*^*3*^*)*	*Increase in nonembolized liver volume (cm*^*3*^*)*	*Changes in hypertrophic ratio (%)*
	γ	Γ	γ	Γ
Total bilirubin (mg/dL)	0.082	− 0.050	− 0131	− 0.102
Prothrombin activity (%)	− 0.039	0.058	0.105	0.145
Albumin (g/dL)	− 0.073	− 0.186	− 0.100	0.036
Total cholesterol (mg/dL)	0.113	0.038	0.105	0.164
CRP (mg/dL)	0.002	− 0.174	− 0.001	− 0.270†
CRP/albumin ratio	− 0.009	− 0.126	− 0.014	− 0.269⁎
Onodera's PNI	− 0.125	− 0.019	− 0.038	0.136
CONUT score at 2 wk	− 0.078	0.040	0.047	− 0.009
Modified GPS at 2 wk	0.093	0.007	0.076	− 0.156
Modified GPS‡				
0	− 50 ± 212	− 82 ± 206	121 ± 107	10.0 ± 6.5
1	− 12 ± 150	− 105 ± 198	164 ± 116	11.2 ± 7.5
2	5.2 ± 322	94 ± 274	148 ± 204	8.1 ± 12.5

⁎ *P* < .05.

† *P* < .01.

‡ Mean ± SD in each score.

### Comparison With Posthepatectomy Complications and In-Hospital Mortality

Table 5 shows the comparison between each correlated parameter with liver volumetric changes during PVE and posthepatectomy morbidity and mortality. In the 64 patients who underwent PVE (Table 5, *A*), LHL15 > 0.92 and increased liver volume > 10% tended to correlate with the lower prevalence of severe complications (Clavien–Dindo grade > IIIa). However, any immunonutritional parameters that correlated with changes in liver volumes after PVE were not significantly associated with posthepatectomy morbidity and mortality. Only increased intraoperative blood loss > 1,500 mL was significantly associated with increased morbidity and mortality (*P* < .05). Although 5 significant continuous parameters were selected using Wilcoxon test and the cutoff data were set using the AUROC analysis for 13 patients without PVE, these were not significantly associated with morbidity after hepatectomy. Mortality was not observed in the 13 patients without PVE. In all 77 patients with or without PVE who underwent hepatectomy, only increased intraoperative blood loss > 1,500 mL was significantly associated with severe morbidity (Clavien–Dindo grade > IIIa) and mortality (*P* < .05).

**Table 5:** Relationship between parameters before and 2 weeks after PVE and posthepatectomy outcomes

	*Clavien–Dindo grade IIIa or more (no/yes)*	P *value*	*In-hospital mortality, (no/yes)*	P *value*
(A) Patients who underwent preoperative PVE followed by hepatectomy (*n* = 64)				
Embolization substance				
Gelatin fragment + coil (*n* = 41)	26/15		39/2	
Iodized oil + Sponzel fragment (*n* = 6)	3/3	.430	5/1	.548
EOI (*n* = 17)	13/4		16/1	
LHL15, < 0.92 (*n* = 19)	13/6		19/0	
≥ 0.92 (*n* = 45)	29/16	.086	41/4	.309
Height, < 155 (*n* = 32)	21/11		30/2	
≥ 155 cm (*n* = 32)	21/11	1.0	30/2	1.0
Total bilirubin, > 0.8 (*n* = 32)	24/8		30/2	
≤ 0.8 mg/dL (*n* = 32)	18/14	.188	30/2	1.0
Prothrombin activity, < 88 (*n* = 25)	16/9		22/3	
≥ 88% (*n* = 39)	26/13		38/1	.291
Total cholesterol,⁎ < 172 (*n* = 28)	17/11		26/2	
≥ 172 mg/dL (*n* = 30)	20/10	.843	28/2	.999
CRP 2 wk after, > 0.27 (*n* = 24)	15/9		21/3	
≤ 0.27 mg/dL (*n* = 40)	27/13	.892	39/1	.144
CRP/albumin ratio at 2 wk, > 0.10 (*n* = 31)	21/10		28/3	
≤ 0.10 (*n* = 33)	21/12	.999	32/1	.339
Changes of whole liver, < 15 cm^3^ (*n* = 37)	27/10		35/2	
≥ 15 cm^3^ (*n* = 27)	15/12	.237	25/2	.999
Decreased liver volume, < 70 cm^3^ (*n* = 28)	17/11		26/2	
≥ 70 cm^3^ (*n* = 36)	25/11	.642	34/2	.999
Increased liver volume, ≥ 130 (*n* = 38)	29/9		37/1	
< 130 cm^3^ (*n* = 26)	13/13	.056	23/3	.358
Hypertrophic liver ratio, < 10 (*n* = 62)	42/20		58/4	
≥ 10% (*n* = 2)	0/2	.115	2/0	.999
Intraoperative blood loss, < 1500 (*n* = 37)	32/5		37/0	
≥ 1500 mL (*n* = 27)	10/17	<.01	23/4	.029
(B) Patients without PVE who underwent scheduled hepatectomy (*n* = 13)				
Age, < 70 y (*n* = 32)	7/1			
≥ 70 y (*n* = 32)	4/1	.999		
Hyaluronate, < 125 ng/mL (*n* = 10)	9/1			
≥ 125 ng/mL (*n* = 3)	2/1	.318		
ICGR15, < 12% (*n* = 10)	9/1			
≥ 12% (*n* = 3)	2/1	.423		
RDW, < 14% (*n* = 8)	8/0			
≥ 14% (*n* = 5)	3/2	.128		
ALT, < 40 IU/L (*n* = 6)	4/2			
≥ 40 IU/L (*n* = 7)	7/0	.192		
(C) All patients who underwent scheduled hepatectomy (*n* = 77)				
Height, < 155 cm (*n* = 40)	26/14		38/2	
≥ 155 cm (*n* = 37)	27/10	.611	35/2	.999
ALT, < 40 IU/L (*n* = 41)	28/13		39/2	
≥ 40 IU/L (*n* = 36)	25/11	.999	34/2	.999
Intraoperative blood loss, < 1500 mL (*n* = 42)	35/7		42/0	
≥ 1500 mL (*n* = 34)	18/17	<.01	30/4	.036

The cutoff for the continuous data was calculated via AUROC analysis.

⁎ The missing value existed in the serum total cholesterol level of 6 patients.

## DISCUSSION

Following PVE, previous reports including our studies have shown that the morphological or functional volumes of the embolized and nonembolized liver change after a few weeks [9,17,20]. Thus, PVE has been established as the standard option to extend the operative indication for chronically injured liver patients [5,6,8,9]. These alterations in both the embolized and nonembolized lobe were also observed in the background of impaired liver in a previous study [17,20]. Moreover, the prediction of hypertrophic volume or rate cannot be estimated with the calculated formula using associated pre-PVE parameters [29,30]. Thus, we supposed that other unknown associated parameters must be examined to understand the mechanism of hypertrophic alteration and to predict volumetric changes. The immunonutritional parameters are closely associated with tissue regeneration and wound healing; therefore, it is hypothesized that changes in hepatic volume would be associated with host production reserves [16]. Each hepatic cell showed an increase in DNA content in a hypertrophic liver [31], which is supposed to be influenced by the energy charge, accelerated growth factors, or adverse inflammatory responses [32,33].

The procedures that yielded the present results were performed through various radiological embolization procedures at the 2 institutes. However, operative indications including the PVE strategy were similar. Thus, the increased hypertrophic ratio was approximately 9% in the present results, which is also similar to the findings of previous reports [17,34]. The 13 cases who underwent major hepatectomy more than 60% without PVE showed good liver function and sufficient estimated volume of remnant liver. Therefore, posthepatectomy outcomes were significantly better than those in the PVE series. Patients who underwent preoperative PVE had borderline results of liver function and estimated liver volumes [35]. Total bilirubin alone would be associated with selection of PVE in the present study. At this point, total bilirubin < 2 mg/dL was generally recognized as an indication for hepatectomy in patients with biliary cancers with obstructive jaundice [6]. A further decrease in hyperbilirubinemia would be necessary to define the adequate indication for patients with 2 mg/dL of bilirubin. Among the embolized materials, EOI did not show an embolization effect. However, the hypertrophic ratio of nonembolized liver was not different when compared with that obtained using other materials. The decrease in the embolized liver volume might not always correlate with hypertrophy per this result, and it is speculated that regeneration or proliferative trigger for stimulation of hepatic cells in the nonembolized liver itself is necessary [36].

Based on these findings of PVE effects in the present study, we analyzed the immunonutritional parameters previously introduced as preoperative risk parameters [12,13,[23], [24], [25], [26], [27], [28]]. Some of these were single parameters, and others were presented as the ratio of 2 combined parameters as shown in the tables. Onodera's PNI, CONUT, and modified GPS are comprehensive scoring systems significantly associated with malnutrition status [37,38] and operative risk including that in liver surgery [12,26,27]. We hypothesized that these immunonutritional parameters were significantly associated with PVE effects, and we planned to devise a calculation formula in multivariate analysis. Based on previous studies, the liver functional reserves safely recover after 2 weeks following PVE [5,6,8,9,20]. Our present study showed that nutritional parameters such as serum albumin and total cholesterol levels significantly decreased; inflammatory parameters such as CRP increased; and comprehensive scores worsened as indicated by an increased CRP/albumin ratio, a decreased PNI, an increased CONUT, and an increased GPS. Two weeks after PVE, some cases showed decreased immunonutritional function. Thus, the scheduled hepatectomy needs to be delayed until these parameters improve. So far, we did not consider this point carefully.

In patients who underwent PVE, significantly correlated parameters were different between the embolized and nonembolized liver in the present study. LHL15, patient height, and total bilirubin level only significantly correlated with decreased volume of the embolized liver. This study included biliary malignancies with obstructive jaundice, and biliary drainage of the diseased liver was not usually performed [39]. Therefore, LHL15 and the total bilirubin level might have been influenced. Although the liver volume is supposed to correlate with patient body weight, body mass index, or physique [40,41], it cannot be explained whether height is related to liver volume. Only prothrombin activity and total cholesterol level significantly correlated with increased liver volume and ratio in the nonembolized liver. Other immunonutritional parameters were not well correlated. Both parameters showed liver activity [42], and these results are understandable. We also examined the relationship among the parameters 2 weeks after PVE and changes in liver volume. As a result, the CRP level and CRP/albumin ratio at 2 weeks negatively correlated with hypertrophy of the embolized liver. CRP is a well-known inflammatory parameter [43], and the excessive or remnant inflammatory status appeared to affect liver hypertrophy. A previous report showed that the CRP or CRP/albumin level was associated with tissue regeneration [44,45]. After PVE, higher levels of CRP in comparison with those pre-PVE would be a surrogate marker for estimating PVE effects and the schedule of hepatectomy. Although pre-PVE chemotherapy may influence the PVE effect, only 2 of 93 patients underwent chemotherapy for colorectal metastasis, and this effect could not be examined in our series. Conversion chemotherapy would be increased in the future era, and influences of chemotherapy-induced liver damage must be investigated in patients undergoing PVE.

The lower LHL15 and hypertrophy degree tended to correlate with severe complication and mortality. Moreover, the increased intraoperative blood loss alone was a significant associated factor in patients undergoing not undergoing preoperative PVE. Previous reports showed that increased blood loss significantly influenced postoperative patient outcomes [45,46]. Increased blood loss > 1,500 mL and associated blood transfusion were independent risk factors per our previous study as well [47]. Considering the associated parameters with increased blood loss in the present results, major hepatectomy must be carefully decided even though preoperative PVE is performed.

This study has some limitations. First, the study is retrospective and used data from a historical cohort from 2 institutes. Although the first author managed these 2 institutes, the prediction of PVE effect remains unclarified. It is difficult to predict the hypertrophic ratio after PVE using conventional serological or liver functional parameters. Although measurement of the molecular alterations or cytokine level is a useful candidate [48], these are difficult to measure in clinical settings. PVE with permanent embolization substances is also challenging to use when considering excessive liver injury. The level of prealbumin, a hepatic protein, is a sensitive parameter of assessing the severity of illness resulting from malnutrition in patients who are critically ill or have a chronic disease, which may reflect the nutritional situation and liver generation [49,50]. However, these parameters have not been fully measured in the present series, and this examination is often difficult under the usual health insurance assessment in our region. Some were examined, but there were many missing values for the present analysis.

In conclusion, we reported the relationship between immunonutritional parameters as operative risk factors and changes in morphological hepatic volume before and after PVE in patients scheduled for major hepatectomy. Contrary to our hypothesis, the candidate parameters were not significantly associated with changes in liver volume. However, the prothrombin activity, total cholesterol level, post-PVE CRP, and CRP/albumin level correlated with hypertrophy in the nonembolized liver. Based on the present data, the scheduled hepatectomy must be delayed until recovery of normal parameters or increased hypertrophy of estimated remnant liver volume in case the inflammatory status persisted a few weeks after PVE.

## Author Contribution

The entire management and evaluation of this program were performed by Professor and Director AN, and he was responsible for the initial conception and design, statistical analysis, writing the article, and the role of critical revision of the article. YT collected patient data at Nagasaki university hospital. KY, MH, NI, TH, KK, EK, YS, KT, and FK contributed performing PVE and collected patient data at Miyazaki University Hospital. TN is an organizer who provided data at Nagasaki University Hospital to AN. Each author certifies approving the manuscript submission.

## Funding Source

This study did not receive any financial support.

## Ethics Approval Statement

All patients' in-hospital data were retrospectively and consecutively collected from patient charts at the 2 institutions. The study design was approved by the Ethics Review Board of NUGSBS and UoM (assigned as #21122008, December 27, 2021, and #O-1034, October 25, 2021, respectively). Informed consent was obtained via an opt-out procedure at an outpatient clinic and on our website information for a month.

## Disclosure

The authors (Atsushi Nanashima, Yukinori Tanoue, Koichi Yano, Masahide Hiyoshi, Naoya Imamura, Takeomi Hamada, Kengo Kai, Eiji Kitamura, Yasuto Suzuki, Kousei Tahira, Fumiya Kawano, Takeshi Nagayasu) declare no proprietary or commercial interest in any product mentioned or concept discussed in this article. No conflict of interest.
